# Tincture of Veratrum Viride

**Published:** 1857-02

**Authors:** 


### EDITORIAL.

*Tincture of Veratrum Viride.*

One or two subscribers have written for instruction in relation to the best mode of preparing this tincture. So far as we know, the method of preparing it does not differ from that of any other alcoholic tincture of a vegetable nature. *Eight* ounces of the dried root should be bruised and put into a bottle, and one pint of alcohol added. It should be allowed to macerate about two weeks, shaking it up well every day. The want of uniformity of strength in the different specimens of tinct. of verat. viride, is not owing so much to the *mode* of preparation as to the quality of the root used. This latter varies chiefly from two causes, viz.:—The length of time the root has been kept after removal from the earth, and the season of the year in which it was dug. The season when the root contains most of its active properties, and consequently the time when it ought to be dug, is in the early autumn, when the leaf just begins to dry. The sooner it is used for preparing the tincture the better, as its properties evidently diminish by age, unless more pains are taken to protect and preserve it than is customary with druggists.

